# Multi-Compartment Lymph-Node-on-a-Chip Enables Measurement of Immune Cell Motility in Response to Drugs

**DOI:** 10.3390/bioengineering8020019

**Published:** 2021-01-31

**Authors:** Nicholas Hallfors, Aya Shanti, Jiranuwat Sapudom, Jeremy Teo, Georg Petroianu, SungMun Lee, Lourdes Planelles, Cesare Stefanini

**Affiliations:** 1 Healthcare Engineering Innovation Center, Biomedical Engineering Department, Khalifa University of Science and Technology, Abu Dhabi P.O. Box 127788, United Arab Emirates; nicholas.hallfors@ku.ac.ae (N.H.); aya.shanti@ku.ac.ae (A.S.); sung.lee@ku.ac.ae (S.L.); 2 Laboratory for Immuno Bioengineering Research and Applications, Division of Engineering, New York University Abu Dhabi, Abu Dhabi P.O. Box 129188, United Arab Emirates; jiranuwat.sapudom@nyu.edu (J.S.); jeremy.teo@nyuad.edu (J.T.); 3 Department of Mechanical Engineering, New York University, P.O. Box 903, New York, NY 10276-0903, USA; 4 College of Medicine and Health Sciences, Khalifa University of Science and Technology, Abu Dhabi P.O. Box 127788, United Arab Emirates; Georg.petroianu@ku.ac.ae; 5 Khalifa University’s Center for Biotechnology, Abu Dhabi P.O. Box 127788, United Arab Emirates

**Keywords:** lymph node-on-a-chip, hydroxychloroquine, motility, rotational motion, reactive oxygen species

## Abstract

Organs On-a-Chip represent novel platforms for modelling human physiology and disease. The lymph node (LN) is a relevant immune organ in which B and T lymphocytes are spatially organized in a complex architecture, and it is the place where the immune response initiates. The present study addresses the utility of a recently designed LN-on-a-chip to dissect and understand the effect of drugs delivered to cells in a fluidic multicellular 3D setting that mimics the human LN. To do so, we analyzed the motility and viability of human B and T cells exposed to hydroxychloroquine (HCQ). We show that the innovative LN platform, which operates at a microscale level, allows real-time monitoring of co-cultured B and T cells by imaging, and supports cellular random movement. HCQ delivered to cells through a constant and continuous flow induces a reduction in T cell velocity while promotes persistent rotational motion. We also find that HCQ increases the production of reactive oxygen species in T cells. Taken together, these results highlight the potential of the LN-on-a-chip to be applied in drug screening and development, and in cellular dynamics studies.

## 1. Introduction

The pharmaceutical drug development industry faces high attrition rates for novel drug candidates [1,2,3]. The concluding statement from an intra-establishment study, involving major pharmaceutical companies AstraZeneca PLC. (Cambridge, UK), Eli Lilly Inc. (Indiana, IN, USA), GlaxoSmithKline PLC. (Brentford, UK) and Pfizer Inc. (New York, NY, USA), is that while improved analytical methods and experimental methodologies have enhanced drug metabolism and pharmacokinetic profiles, failures due to efficacy and safety have maintained attrition rates [2]. In particular, 1/3 of the failures of new drug candidates are attributed to drug toxicity which, in turn, includes immune toxicity [4].

One major limitation hindering the study and development of immune compatible drugs is the lack of experimental models that fully recapitulate ex-vivo the properties of human immune organs, which are extremely complex in nature [5,6]. Therefore, novel bioengineered platforms and organ-on-chips that closely mimic the native microenvironment of cells should be explored, particularly in the context of less toxic and more efficient drug design.

Recently, microfluidic technology has been utilized in biological research since it allows high throughput studies of cellular systems in in vivo-like microenvironments and enables miniaturization of components [6,7,8]. Using microfluidic technology, several groups have developed ex vivo immune-related organs to further investigate immune mechanisms that participate in major diseases and to develop new therapeutic approaches [8,9,10,11,12,13,14,15]. For instance, Singh et al. generated mouse 3D synthetic immune organoids capable of undergoing germinal center reactions [11,12]. Furthermore, Giese et al. developed in vitro lymphatic micro-organoids and utilized them for immunological drug testing [13,14]. 

Despite the great efforts invested in developing in vitro models of immune organs, the complex architecture of the lymph node (LN) has still not been fully replicated in vitro even though the LN is an organ where key immune-drug interactions take place [6,14,15]. The LN is an organ composed of various immune cell types spatially distributed in different micro-domains and of vessels that collect and filter the draining interstitial lymph that flows across all tissues [16,17]. The structure of the LN is described as a densely packed cellular environment (B lymphocytes, T lymphocytes and macrophages) with an extracellular matrix (ECM) primarily made up of type I collagen [18,19]. Lymph enters the LN through afferent vessels into the subcapsular sinus (SCS), characterized by the presence of macrophages that play a crucial role in capturing, delivering and presenting antigens [20,21]. As lymph flows through the SCS, a fraction of the fluid gets diverted laterally into a web-like conduit system formed by reticular collagen fibrils that penetrate the B cell follicle as well as the paracortex where the T cells are located [22]. This unique structural microenvironment of the LN facilitates the optimal activation, proliferation, and differentiation of the lymphocytes in order to trigger appropriate immune responses towards toxic agents and infections [23].

In an effort to contribute to drug discovery, our lab has developed a LN on-a-chip that incorporates key features of the native human LN including, (i) comparable extracellular matrix composition, morphology, porosity, stiffness, and permeability, (ii) compartmentalization of immune cells within distinct structural domains, (iii) replication of the lymphatic fluid flow pattern, (iv) viability of encapsulated cells in hydrogel over the typical timeframe of immunotoxicity experiments, and (v) interaction among different cell types across chamber boundaries [24,25]. To the best of our knowledge, there is no readily available in vitro system of the LN that nears the level of biomimetics achieved by our LN-on-a-chip, namely the 3D reconstruction of the LN microenvironment. This artificial biomimetic LN is an attractive alternative to further dissect and understand the mechanisms of action of drugs delivered to cells in a context that nears the in vivo LN microenvironment more than the existing state-of-the art. The LN-on-chip is intended for identifying comparative behavior of immune cells in response to drugs and for recognizing patterns of action. 

Hydroxychloroquine (HCQ; molecular formula: C_18_H_26_ClN_3_O) is a derivative of chloroquine with immunomodulatory properties, that has been FDA-approved for the treatment of numerous inflammatory and autoimmune conditions, including malaria, lupus, and rheumatoid arthritis [26]. HCQ has recently attracted attention in the framework of the ongoing pandemic, leading to dozens of clinical trials [27,28]. It has been described that HCQ interferes with lysosomal activity and autophagy, interacts with membrane stability and alters signaling pathways and transcriptional activity [29]. However, despite an abundance of empirical data, the mechanisms involved in the immunomodulatory activity of HCQ have not been fully characterized. 

In the present study, the LN-on-a-chip is used as a model to investigate cell dynamics in response to HCQ. Cellular motility is essential in the lymph node not only because lymphocytes circulate in and out this immune organ but also because T and B cells need to move inside the lymph node in order to generate a proper immune response [30,31]. Lymphocytes move for searching and presenting antigens, for interacting with other cells and for proliferating. Inside the lymph node, naïve T cells have been described to move by a random walk behaving as sentinels looking for foreign antigens [32,33]. We selected Jurkat T and Raji B cells because they are, respectively, classical T and B cellular models in immunology, extensively used during the last 20 years to characterize the biology and function of T and B lymphocytes [34,35,36,37,38,39,40,41,42,43,44,45,46,47,48,49,50,51,52]. Indeed, Jurkat T and Raji B cells have been used to dissect and understand the molecular mechanisms behind cell polarization and immunological synapse, cell movement and activation, antigen presentation, and also to study drug responses [34,35,36,37,38,39,40,41,42,43,44,45,46,47,48,49,50,51,52,53]. T cells and B cells belong to the same lymphoid lineage but differ in multiple aspects such as their site of maturation, surface receptors, and their function during the immune response [54]. They are the main cellular components of the lymph node; thus, we included both of them in the device, in order to approach the in vivo situation. Activity and efficacy of immune competent cells depend on a number of factors such as their ability to move and their ability to generate reactive oxygen species (ROS). The purpose of the present work was to establish base line values for these essential parameters and to compare and contrast with the effect of exposure to clinically relevant concentrations of a known immune-modulator i.e., HCQ. We found that HCQ acts on the migratory machinery of T cells; it reduces cellular velocity while favoring persistent rotational motion (PRM). Moreover, we showed that levels of cellular ROS increase after HCQ treatment in T cells which could partly explain the changes observed in motility. In contrast, no significant changes in motility and ROS where detected with Raji B cells.

## 2. Materials and Methods

### 2.1. LN-on-a-Chip Design and Fabrication 

**Figure 1:**
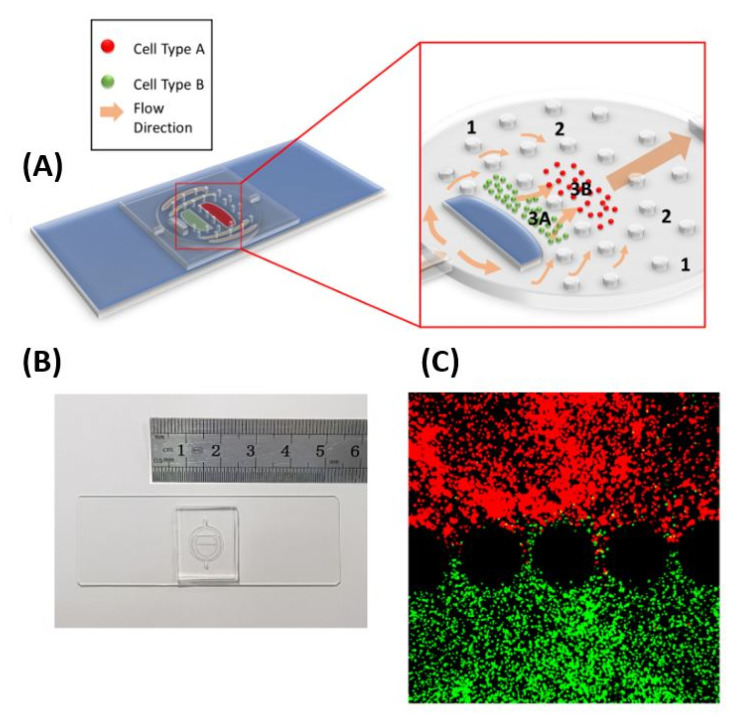
Lymph Node-on-a-chip (LN-on-a-chip). (**A**) Schematic showing the different regions of the device formed using micropillars. Region labelled 1 corresponds to the Subcapsular Sinus of the native LN, Region labelled 2 corresponds to the Reticular Network, Region labelled 3A corresponds to the Follicle and Region labelled 3B corresponds to the Paracortex. Regions 3A and 3B are cellular regions loaded with immune cells embedded in 3D hydrogel matrices. (**B**) Miniaturized LN-on-a-Chip. With respect to the previous published device, the LN-on-a-chip has been further miniaturized (2.5 times smaller) to allow efficient and high throughput experimentation and (**C**) two different fluorescently labelled immune cells (Raji B-red and Jurkat T-green), each in a distinct compartment within the LN-on-a-Chip.

As described in a previous publication, the developed LN-on-a-chip consists of a body, an inlet and an outlet [24]. The body is further divided into an outermost region corresponding to the subcapsular sinus of the native LN, a middle region corresponding to the reticular conduit network of the native LN, and an inner region corresponding to the cellular regions of the native LN as shown in Figure 1A. In particular, the inner region is divided into two sub-regions; one hosts T cells and corresponds to the paracortex of the native LN and one hosts B cells and corresponds to the follicle of the native LN. All regions are separated from one another using circular micropillars, which allow for the confinement of extracellular matrix components within specific compartments.

To realize an enabling technology, further miniaturization of the device design and thus increasing throughput have been accomplished (Figure 1). In particular, device schematics were drafted in AutoCAD 2018 (AutoDesk Inc., San Rafael, CA, USA), and photomasks were produced by Front Range Photomask (Lake Havasu, AZ, USA). A DRIE Bosch process (Plasmalab 100, Oxford Instruments, Abingdon, UK) was used to etch 400 micron deep trenches into silicon wafers (University Wafer, Boston, MA, USA) to produce the mold. PDMS elastomer (Corning Inc., Corning, NY, USA) was poured into the mold, and cured at room temperature for several days, followed by 2 h at 115 °C to remove residual monomer. After curing, devices were manually separated with a blade. Subsequently, oxygen plasma bonding was performed (Plasma prep III, Structure Probe Inc., West Chester PA, USA) at 1000 mTorr and 20 watts to permanently bond the chips to glass microscope slides (Corning) to produce the finished LN device. Plasma surface treatment leaves the inner device walls hydrophilic, preventing the adsorption of hydrophobic elements from cell media.

Deep reactive ion etching resulted in 400 micron deep features on the mold. A thick layer of photoresist was sufficient as an etch mask. After etching features and stripping resist, a brief exposure to C_4_F_8_ plasma created a thin hydrophobic film, easing removal of PDMS. The etched silicon structures, treated with a hydrophobic coating are suitable for long term, repeated molding. The deep etches resulted in sufficiently tall features for a 10 microliter total volume within the device. This volume can accommodate roughly 30,000 cells at 2,000,000 cells/mL, enough for statistically significant analysis of cell behavior, while also allowing single cell tracking and analysis if so desired. Plasma bonding effectively sealed the device, creating a water tight boundary between the chip and glass. Even contact between glass and PDMS indicates a highly anisotropic silicon etch with good uniformity.

### 2.2. Cell Culture

In this study, Jurkat T cells (ATCC, Manassas, VA, USA) and Raji B cells (AddexBio, San Diego, CA, USA) were used. Jurkat T cells are cells derived from acute human (male) T cell leukemia and Raji B cells are lymphoblast-like cells established from a male Burkitt’s lymphoma. Cells were cultured, as recommended, in Roswell Park Memorial Institute (RPMI) 1640 medium containing 4.5 g/L D-glucose (hyperglycemic amount), 2.383 g/L 4-(2-hydroxyethyl)-1-piperazineethanesulfonic acid (HEPES), 0.3 g/L L-glutamine, 1.5 g/L sodium bicarbonate, and 0.11 g/L sodium pyruvate (Gibco, Fisher Scientific, Waltham, MA, USA). HEPES buffer was required to maintain the physiological pH while preparing the cells in the device and during live imaging. The media was supplemented with 10% heat inactivated fetal bovine serum (FBS; Gibco) and 1% penicillin–streptomycin (Biosera, Nuaille, France). The cells were incubated at 37 °C and 5% CO_2_.

### 2.3. Cell Staining

For some of the time-lapse microscopy experiments, Raji B and Jurkat T cells were labelled in order to be distinguishable from one another while under the microscope. Cells were stained using CellTracker (Invitrogen, Thermo Fischer Scientific) dyes following the manufacturer’s protocol. In brief, a CellTracker stock solution was prepared by diluting the product in dimethyl sulfoxide (DMSO) (Sigma-Aldrich, St. Louis, MO, USA) to a final concentration of 10 mM. Next, a staining working solution was prepared by diluting the stock solution to a final concentration of 1 μM in RPMI serum-free media. Cells were then harvested, suspended in pre-warmed working solution and incubated for 30 min at 37 °C and 5% CO_2_. Finally, the staining solution was removed from the cells and they were resuspended in full growth medium and used for experiments within 1–3 days. Jurkat T cells were stained green while Raji B cells were stained red.

### 2.4. Gel Protocol and Device Filling

Cells at concentration of 2–3 × 10^5^ cells/mL were suspended in collagen gel (Gibco, Fisher Scientific) at 2 mg/mL in phosphate buffer at pH 7.5 as described by Sapudom et al. [55]. A 250 micron glass capillary coupled to a GasTight syringe (Hamilton Co., Reno, NV, USA) was used to specifically fill the separate chambers with the desired cell populations (around 2–3 × 10^4^ cells). Filled devices were then incubated for 30 min for polymerization of the collagen gel.

### 2.5. Time Lapse Experiments and Cell Tracking Analysis

To analyze the motility of immune cells within the LN-on-a-Chip, we loaded the different cell types into their designated chambers and mounted the device on a microscope stage with a built in incubator for live cell tracking (Zeiss AxioCam HRm, Carl Zeiss, Oberkochen, Germany). We took images of the cells every 90 s for 1–3 h depending on the study and we continued to perfuse the system with media (flow rate 3 μL/min) for the whole duration of imaging using a microfluidic pump (PHD Ultra, Harvard Apparatus, Massachusetts, MA, USA). We tracked the movement of the cells using the tracking plugin provided by ImageJ and a custom built analysis tool [56,57,58]. Microsoft Excel (Microsoft corporation, Washington, WA, USA) was then used to calculate cell motility statistics. Cells were considered motile and included in the analysis when they moved a minimum distance equivalent to a cell body. A minimum of 50 cells were analyzed per experiment. The frequency of motility was calculated as the percentage of motile cells. Coordinate data was imported from the tracking algorithm, and x and y values at each time point were used to calculate total track length, net length and average step sizes. The tracking algorithm returned pixel coordinate values, so all measurements were normalized to micrometers by microscope manufacturer specified pixel to micron ratios. See Figure S1 for detailed Excel algorithms.

To assess the effect of HCQ (Sigma-Aldrich) on cell movement, Jurkat T and/or Raji B cells were loaded in the LN-on-a-chip chambers and HCQ was added in the culture medium and administered through a continuous flow along the experiment. A 5 μM concentration of HCQ was chosen, based on the cytotoxicity study described in Section 2.6. Cellular motility was visualized by time-lapse microscopy and images were analyzed using the tracking plugin provided by ImageJ and a custom built analysis tool [56]. Microsoft Excel was then used to calculate cell motility statistics as described above.

### 2.6. Cytotoxicity of Hydroxychloroquine

To measure the toxicity of HCQ to Jurkat T and Raji B cells, an MTT (3-(4,5-dimethylthiazol-2-yl)-2,5-diphenyltetrazolium bromide) (Sigma-Aldrich) reduction assay was performed. In brief, cells of both types were seeded in 96 well plates at a concentration of 6 × 10^5^ cells/mL and incubated for 24 h at 37 °C and 5% CO_2_. After 24 h of incubation, the drug was added to cells at variable concentrations (5, 20, 50 and 200 μM). Cells with media only and no drug were kept as a control. Cells were then incubated for an additional 48 h. After 48 h, MTT was added to cells and cells were subsequently incubated for 4 h. During this incubation time, yellow MTT solution was reduced to purple formazan in living cells. After 4 h, media containing MTT was removed from all wells and replaced by DMSO. The absorbance of each sample was then measured at 570 nm using the microplate reader. Percentage cell viability was calculated by comparing the absorbance of the control cells (cells with medium only and no drug) to that of cells with the drug. Percentage cell viability = (cells with drug and MTT) / (control cells with MTT but no drug) × 100.

### 2.7. Assessment of Reactive Oxygen Species

To assess whether HCQ affects the production of ROS or not, the amount of ROS produced by cells incubated with HCQ was determined and compared to that of cells incubated with media only using CM-H2DCFA (DCFDA) (Invitrogen, Thermo Fischer Scientific) ROS detection kit. In brief, cells of both types were seeded in 96 well plates at a concentration of 6 × 10^5^ cells/mL and incubated for 24 h at 37 °C and 5% CO_2_. After 24 h of incubation, the drug was added to cells at variable concentrations (5 μM, 50 μM, 200 μM). After 3 h and after 24 h, 2 μM of DCFDA dye was added to the cells and they were incubated for 30 min at 37 °C and 5% CO_2_. After 30 min, the fluorescence of each well was measured at λ_ex_ = 480 nm and λ_em_ = 520 nm using the microplate reader (Tecan Trading AG, Zurich, Switzerland).

### 2.8. Analysis of Cellular Rotational Motility

**Figure 2:**
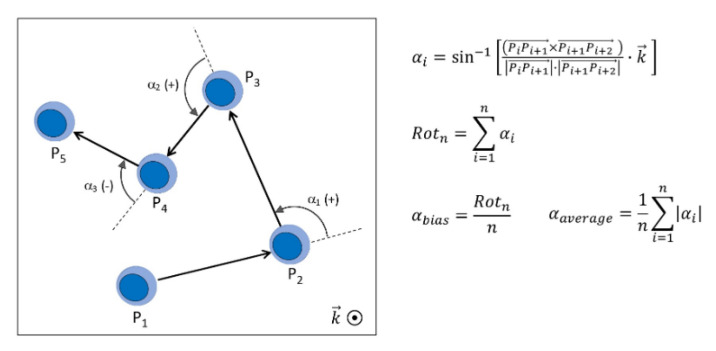
Schematic showing the rotational motion analysis.

We assessed whether HCQ affects the rotational motion of cells. We used the cell tracking information to calculate, per imaging frame, the average angular movement *α_average_*, and the angular bias *α_bias_* that contributes to cumulative rotation *Rot_n_*. With reference to Figure 2, *α_average_* (angular movement) is the magnitude of the average angular movement per frame, which normally occurs in clockwise or counterclockwise direction in a balanced manner, while *α_bias_* (angular bias) is the quantification of the Persistent rotational motion, i.e. the per-frame deviation from a non-rotational motion, either clockwise or counterclockwise. Equations applied are also reported in the figure.

### 2.9. Statistical Analysis

All experiments were done at least in triplicate and data is presented as mean ± standard error mean. Unpaired Student t-tests were used to determine statistical significance of different groups. In all statistical analysis, *p* < 0.05 was considered significant.

## 3. Results

### 3.1. Three-Dimensional Lymph Node-on-a-Chip model to Study Cellular Dynamics

**Figure 3:**
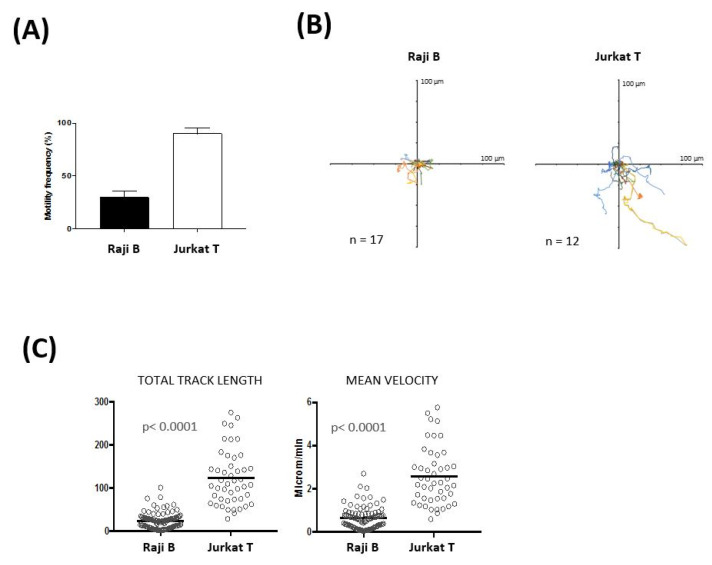
3D Lymph-Node on-a-Chip supports cell motility. (**A**) Frequency of motility (**B**) representative individual cell tracks (**C**), total track length and velocity of Jurkat T and Raji B cells seeded in the LN-on-a-chip. Time-lapse imaging was performed and movie recorded for 150 min. Data in panel A corresponds to the merge of 3 experiments and represents mean ± standard error mean.; tracks plotted in panel B correspond to one representative experiment and data in panel C represent individual cells from three pooled experiments (mean is also indicated).

Trafficking of lymphocytes within lymphoid organs is essential for initiating contact with antigen-presenting cells [30]. Moreover, the ability of B and T cells to move among strategic locations in the LN is critical to achieve a fully humoral response [31]. Two-photon laser microscopy has allowed to characterize B and T cell movement within the LN and has shown that lymphocytes move by a random walk [32,33]. In order to address whether the LN-on-a-chip supports cellular motility we analyzed in real time the movement of T and B immune cells by time-lapse microscopy. Jurkat T cells and Raji B cells were embedded in the collagen gel, injected in the microfluidic flow-through device and monitored for 150 min. We found that 90% of Jurkat T cells while only 30% of Raji B cells move freely inside the biomimetic LN (Figure 3A). The rose plots showing the cellular tracks stress the characteristic random T cell motility (Figure 3B) and underlie the different motility behaviors between the two cell types. Jurkat T cells travel longer distances than Raji B cells (average track length 123.4 ± 9.4 μm for Jurkat T cells and 24.5 ± 1.9 μm for Raji B cells; Figure 3C), and move with an increased average velocity (Jurkat T cells 2.6 ± 0.2 μm/min; Raji B cells 0.66 ± 0.05 μm/min; Figure 3C). Similar speed values have been previously reported for these cells in different experimental settings [59,60]. These results are also consistent with previous studies showing that, in their native environment, T cells move more than B cells (motility coefficient is five to six times bigger) and explore a wider territory [32]. Taken together, these results show that we have established a 3-D LN model that supports immune cell movement.

### 3.2. Impact of Hydroxychloroquine on Translational and Rotational Cell Motility 

**Figure 4:**
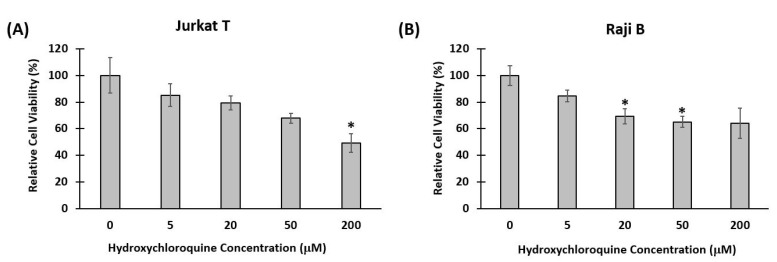
Relative viability of cells treated with hydroxychloroquine. Jurkat T and Raji B cells were incubated with different concentrations of hydroxychloroquine for 24 h and then MTT assay was performed to measure cytotoxicity. (**A**) Viability of Jurkat T cells. At low concentrations of the drug (5, 20, 50 μM), there was no significant difference between the viability of cells treated with HCQ and the viability of control cells. At higher concentration (200 μM), there was a significant decrease in the viability of cells treated with HCQ compared to control cells. (**B**) Viability of Raji B cells. At low concentrations of the drug (20, 50 μM), there was a significant decrease in the viability of cells treated with HCQ and the viability of control cells. (* *p* < 0.05 compared to control- no hydroxychloroquine treatment).

The observation that immune cells move freely in the lymph-node-on-a-chip and that cellular motility can be monitored in a controlled dynamic microsystem led us to address its potential use for drug development. We decided to analyze B and T cell movement in response to HCQ, due its recent introduction as a potential treatment of current SARS-CoV-2 (Cov-19) pandemic, and the fact that the mechanisms involved in its immunomodulatory activity have not been fully characterized [27]. Initially, we performed a toxicity curve (MTT reduction assay) to measure the toxicity of HCQ after it was incubated with Jurkat T and Raji B cells for 24 h. Figure 4 shows the percent viability of both cell types cultured with different concentrations of HCQ. In fact, for Jurkat T cells, at low concentrations of the drug (5, 20, 50 μM), there was no significant difference between the viability of cells treated with HCQ and the viability of control cells. However, at higher concentration (200 μM), there was a significant decrease in the viability of Jurkat T cells treated with HCQ compared to control cells. In fact, we determined the LD50 for Jurkat to be 200 μM. On the other hand, for Raji B cells, even low concentrations of the drug (20, 50 μM), caused a significant decrease in the viability of cells treated with HCQ compared to control cells. A concentration of 5 μM HCQ was selected for cell motility experiments as this concentration is 40 times lower than the LD_50_, has been employed in other studies and is within the accepted limit for therapeutic range [61,62,63]. 

**Figure 5:**
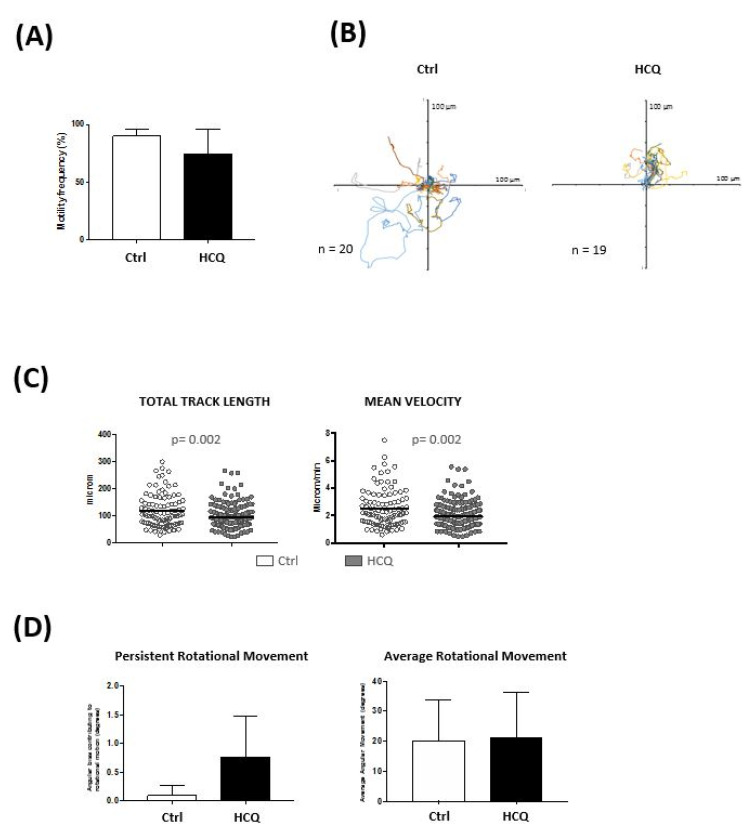
Cell motility in response to Hydroxychloroquine (HCQ). (**A**) Frequency of motility (**B**) representative individual cell tracks (**C**) total track length and velocity and (**D**) persistent rotational motion and average rotational movement per step of Jurkat T cells seeded in the LN-on-a-chip. Hydroxychloroquine was added in the culture media and administered through a continuous. After 30 min of HCQ treatment, time-lapse imaging was performed and movie recorded for 150 min. Data in panel A and D correspond to the merge of three experiments and represents mean ± standard error mean; tracks plotted in panel B correspond to one representative experiment; data in panel C represent individual cells from three pooled experiments (mean is also indicated).

To evaluate the effect of HCQ on cell movement, Jurkat T and Raji B cells were seeded in the microchip chambers and HCQ (5 μM) was added in the culture medium and administered through a continuous flow along the experiment. Cellular motility was analyzed by time-lapse microscopy. We found that Jurkat T cell motility was significantly decreased in the presence of HCQ (Figure 5D). However, no effect was observed in Raji B cells (Figure S2). Total track length was reduced by 21% (average track length 119.7 ± 6.3 μm for control cells and 94.84 ± 3.0 μm for HCQ treated cells, Figure 5D). Similarly, a slower motion was detected in Jurkat T cells in the presence of HCQ (average velocity 2.49 ± 0.13 μm/min for control cells and 1.98 ± 0.06 μm/min for HCQ treated cells, Figure 5D). 

Recently, rotational motion of cells has attracted wide attention in both cellular and developmental biology [64]. Interestingly, cells cultured on 2D substrates do not tend to exhibit rotational motion, but cells cultured on 3D substrates tend to do so [64]. We analyzed the rotational motion of T and B cells in response to HCQ and found that this drug induces persistent rotational motion (PRM) in Jurkat T cells with no change in average angular movement per step. PRM was calculated as the angular bias contributing to the cumulative rotation (PRM 0.097 for control cells and 0.76 for HCQ treated cells, fold change 8, Figure 5D). It has been described that various cell polarity mechanisms regulate persistent rotational motion of mammalian cells [65]. Altogether, our results show that HCQ affects the motility behavior of T cells. Further studies are needed to clarify the mechanisms behind this effect and its biological relevance.

### 3.3. Effect of Hydroxychloroquine on Reactive Oxygen Species Production

**Figure 6:**
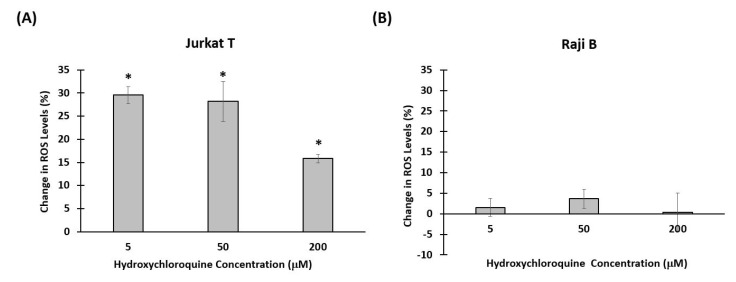
The percent change in Reactive Oxygen Species (ROS) in response to hydroxychloroquine (HCQ). (**A**) Percent change in Jurkat T cells. There was a significant increase in ROS levels in cells treated with HCQ compared to control cells. This increase was seen at all concentrations of the drug (* *p* < 0.05 compared to control- no HCQ treatment) (**B**) Percent change in Raji B cells. There is no significant change between ROS produced by cells treated with HCQ and that produced by control cells.

ROS are generated during mitochondrial oxidative metabolism as well as in response to external signals such as cytokines, and bacterial invasion [66]. ROS regulate many signal transduction pathways and have been involved in multiple immune cell functions including positive responses such as cell activation and movement, but also in negative ones, such as cell death or growth inhibition [67]. Cells need to clear ROS to regulate the reduction-oxidation (REDOX) balance and avoid oxidative stress, which induces progressive cellular damage and has been associated with numerous human pathologies [47,68,69,70]. As chloroquine and hydroxychloroquine induce ROS production in human astrogial cells, we decided to investigate whether ROS might be involved in the motility behavior observed in response to HCQ in our model [71]. We measured the amount of ROS produced by cells after incubation with HCQ for 24 h. Figure 6 shows the percent change in ROS in response to HCQ after 24 h. There was a significant increase in ROS levels in cells treated with HCQ compared to control cells. This increase was seen at all concentrations of HCQ. We also checked the amount of ROS produced by Raji B cells upon exposure to HCQ, but we did not see any significant change between ROS produced by cells treated with HCQ and that produced by control cells. Our results indicate that HCQ stimulates ROS production in immune T cells at a concentration currently used in clinic. It would be relevant to investigate the implication of HCQ-induced ROS in T cell functionally and address its involvement in the motility response we observed. 

## 4. Discussion

The complexity of interaction between a drug and the different immune cell types is not easy to be replicated with conventional cell culture plates. Thus, more sophisticated and more physiologically relevant in vitro systems are required. In this study, we sought to demonstrate the ability of our newly developed LN-on-a-chip to support cellular motility and immune-drug interaction studies in an in vivo like LN microenvironment using HCQ as a model drug. When administered, HCQ is found widely distributed in the human body accumulating in tissues and blood cells [72,73,74,75,76]. We show, through continuous real time monitoring, that T and B immune cells freely move in the biomimetic LN-on-a-chip. HCQ negatively affects the translational motility of T cells while induces rotational motion of the cells. We hypothesize that this change in rotational motion is partly due to increased production of ROS in response to HCQ exposure.

From a technical perspective, the LN-on-a-chip is well suited for efficient, high throughput experimentation. Device features are 400 microns tall, for a total chamber volume of 10 microliters. The low height of the chamber allows for proper imaging of cells despite the opacity of the gel, which rapidly increases with thickness. Additionally, very low volumes of cells and reagents are needed to fill the device allowing for highly efficient use of cells and reagents. Wafer layout allows for the molding of multiple devices simultaneously, leading to high throughput chip production with little waste. A highly scalable production process, combined with very low reagent utilization signify that this system can be deployed for large-scale studies in an efficient and highly repeatable fashion. The device supports long term culturing of cells and our data show that the device supports in-situ viability testing (Figure S3) providing a user-friendly method of measuring cell death without the need to extract cells from the device.

Our developed LN-on-a-chip consists of multiple components that make it superior to 2D cell culture plates and more suitable for immune-drug interaction studies and these include (1) multi-compartments that allow co-culture of multiple immune cell types simultaneously (2) 3D biomimetic matrices that resemble the ECM components of the native human LN and (3) fluid flow that is similar to that of the native human LN. The LN-on-a-chip is transparent and thus enables real-time monitoring of multiple immune cell types at the same time under the microscope. Time-lapse microscopy showed that Jurkat T and Raji B cells are freely moving in the LN-on-a-chip. Jurkat T cells show higher motility than Raji B cells, with longer cell trajectories and increased speed, in agreement with the behavior observed in the human LN [32]. Next experiments including primary T and B cells will help us to confirm our current results and to demonstrate the suitability of the platform to support cell motility in closer conditions to the in vivo situation.

Cellular movement is an essential process to all living things as it enables the development of an organism (morphogenesis), the healing of wounds and the response to foreign antigens [77]. Movement among different cellular types is extremely diverse and depends on their physiological state; healthy or diseased [78]. In the lymph node cellular motility is essential to generate a proper immune response; T and B lymphocytes are found in specific locations and need to move for searching and presenting antigens, for interacting with other cells, for proliferating among others [30,31]. In 3D models based on collagen matrix, it has been reported that focal adhesion is the primary mechanism by which cells interact with the extracellular matrix (ECM) and that vinculin regulates cell polarization and directionality [79]. The LN-on-a-chip here described would be a suitable model to continue these studies and investigate the mechanisms behind cell–matrix adhesion and signaling that regulate cell polarity and movement. Moreover, it could be used to model more complex immune reactions in which cell motility is essential such as antigen presentation, cell activation and antibody production [30,31]. Exploring these opportunities might lead to a novel and attractive platform for vaccine and drug development. 

Besides the translational movement, cells cultured on 3D substrates are able to rotate. Epithelial and endothelial cells have been shown to undergo persistent rotational motion around each other in micro-patterned cell culture plates [65]. Rotational motion has been related to collective cell motility and might be involved in processes such as cancer spreading and embryo development [65]. Here, we observed that HCQ reduces translational movement while induces persistent rotational motion in T cells. It has been proposed that different polarity mechanism including contact inhibition of locomotion and neighbor alignment regulate persistent rotational motion [65]. Further studies on rotational motility using primary cells in the LN-on-a-chip will help to evaluate the significance of our observation and the mechanism underlying. One possibility is that HCQ interferes with cell’s internal chemical polarity.

We have also found that HCQ induces ROS production in T cells which might collaborate in the increased rotational motion detected. A growing body of evidence suggests that ROS play a key role in regulating cellular movement. We hypothesize that in our model, ROS production might induce rotational motion but it is not sufficient to promote translational cell motility; other pathways activated by HCQ would be contributing to the reduced cell motility. Further studies using primary T and B cells are needed to confirm these results and address its relevance. Our results highlight the potential of the LN-on-a-chip as a novel 3D platform to study cellular dynamics and complex biological processes as well as to assess drug-cell interactions in a multi-compartment and user-friendly system.

## Data Availability

The data presented in this study are available on request from the corresponding author.

## Supplementary Materials

The following are available online at https://www.mdpi.com/2306-5354/8/2/19/s1, Figure S1: Cell tracking analysis, Figure S2: HCQ does not interfere with Raji low motility, Figure S3: Cell toxicity in situ in the LN-on-a-chip device.
