# Supplementary material for: Multi-Compartment Lymph-Node-on-a-Chip Enables Measurement of Immune Cell Motility in Response to Drugs
Source: Bioengineering (Basel). 2021 Jan 31;8(2):19. doi: 10.3390/bioengineering8020019 (PMC7912616; doi:10.3390/bioengineering8020019)
Supplement: Supplementary file 1 [file bioengineering-08-00019-s001.pdf]

Supplementary Materials

Multi-Compartment Lymph-Node-On-A-Chip Enables Measurement of Immune Cell Motility in Response to Drugs

Nicholas Hallfors [1], Aya Shanti [1], Jiranuwat Sapudom [2], Jeremy Teo [2,3], Georg Petroianu [4], SungMun Lee [1,5], Lourdes Planelles [1,*] and Cesare Stefanini [1,*]

1 Healthcare Engineering Innovation Center, Biomedical Engineering Department, Khalifa University of Science and Technology, Abu Dhabi, P.O. Box 127788, UAE
2 Division of Engineering, New York University Abu Dhabi, Abu Dhabi, P.O. Box 129188, UAE
3 Department of Mechanical Engineering, New York University, New York City, NY 10276-0903, P.O. Box 903, USA
4 College of Medicine and Health Sciences, Khalifa University of Science and Technology, Abu Dhabi, P.O. Box 127788, UAE
5 Khalifa University's Center for Biotechnology, PO Box 127788, Abu Dhabi, United Arab Emirates
* Correspondence: cesare.stefanini@ku.ac.ae, +9712-501-8472, maria.carazo@ku.ac.ae, +9712-501-8472

1) Raw Data (X,Y)
   a. Step size $(dX_i) = X_i - X_{i-1}$
   b. Step size $(dY_i) = Y_i - Y_{i-1}$
      i. Step length $l_i = \sqrt{dX_i^{\,2} + dY_i^{\,2}}$ → Track length = $\sum_{i=1}^{n} l_i$
      ii. Net distance $d = \sqrt{(X_n - X_1)^2 + (Y_n - Y_1)^2}$

      Velocity = Track length / time

Supplementary Figure 1. Cell tracking analysis

Excel algorithms used to calculate cell motility statistics. Coordinate data was imported from the tracking algorithm, and x and y values at each time point were used to calculate total track length, and velocity. The tracking algorithm returned pixel coordinate values, so all measurements were normalized to micrometers by microscope manufacturer specified pixel to micron ratios.

Figure S1.

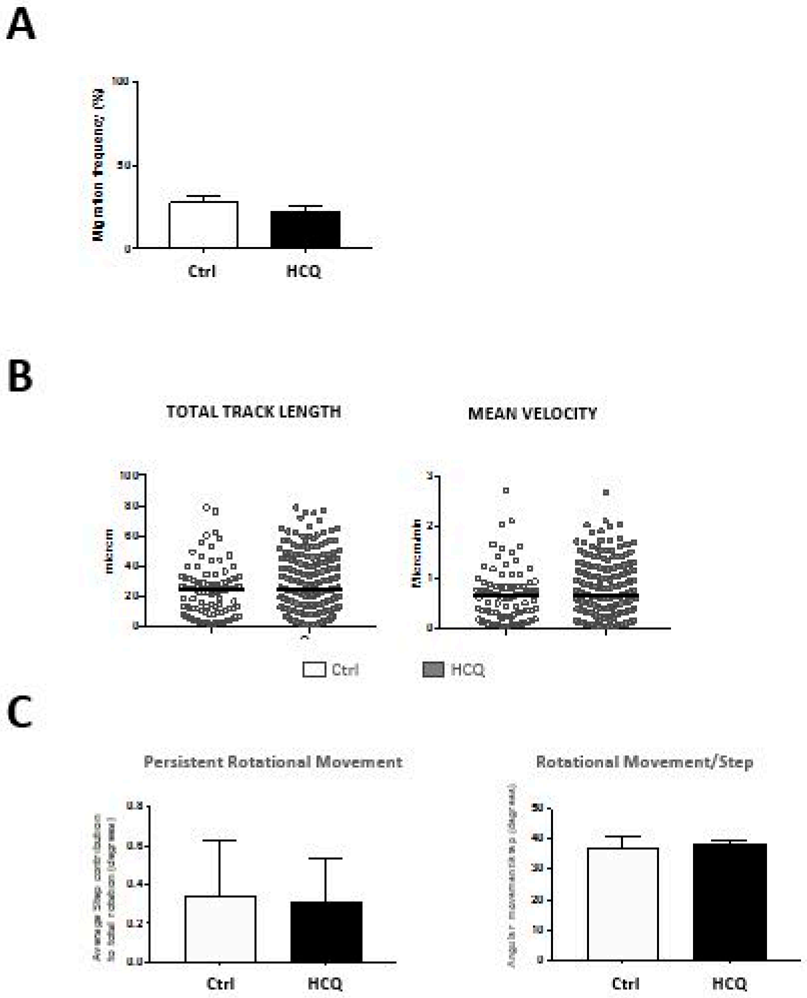

Supplementary Figure 2: HCQ does not interfere with Raji low motility
Frequency of migration (A), total track length and cell velocity (B), and persistent rotational motion and rotational movement per step (D) of Raji B cells. Raji cells (2 x10^6 cells/ml). were seeded in the LN-on-a-chip, hydroxychloroquine (5 μM) was added in the culture media and administered through a continuous flow as described in *Mat and Meth* section. After 30 minutes of HCQ treatment, time-lapse imaging was performed and cell movement was video recorded for 150 minutes. Data in panel A and C correspond to the merge of 3 experiments and represent average +_ SEM; data in panel C represent individual cells from three pooled experiments (mean is also indicated). at least 50 cells were analyzed per experiment;

Figure S2.

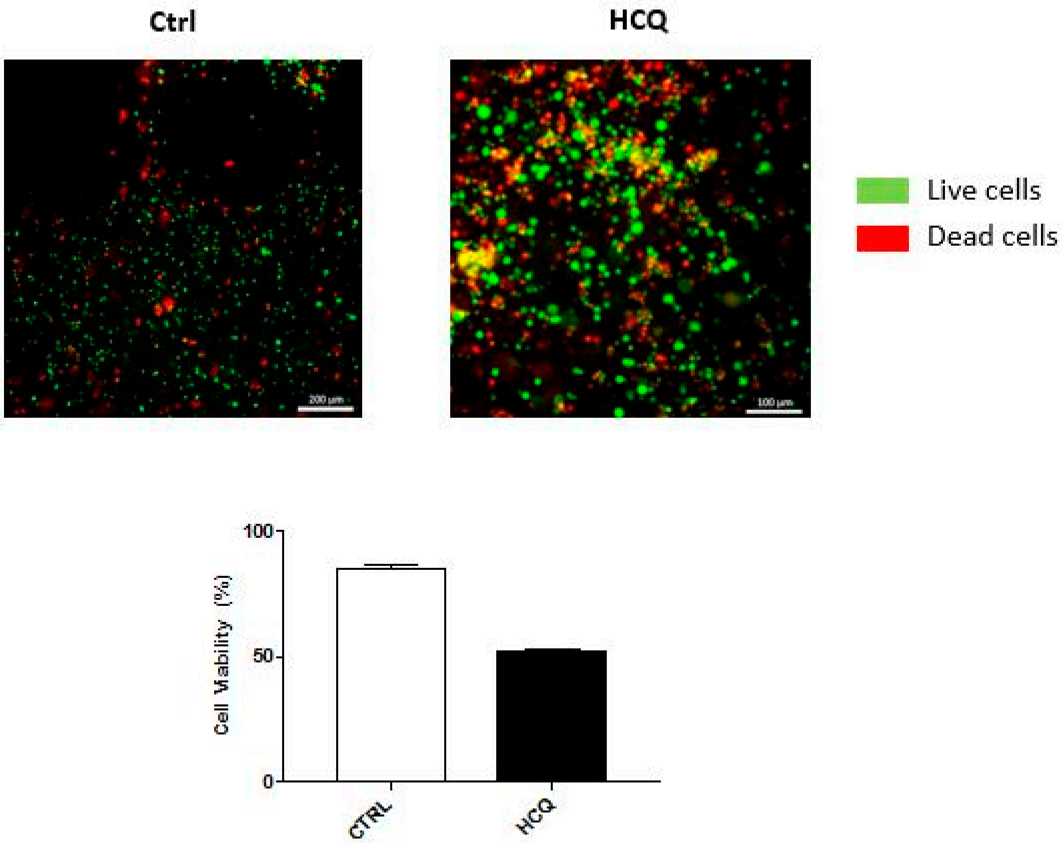

Supplementary Figure 3. Cell toxicity *in situ* in the LN-on-a-chip device

Jurkat T cells ($2x10^6$ cells/ml) were seeded in the LN-on-a-chip. Hydroxychloroquine (200 µM) was added in the culture media and administered through a continuous flow during for 20 hours as described in Materials and Methods. After 20 hours, dies from live/dead kit (Invitrogen, Thermo Fischer Scientific, Waltham, MA, USA) were included in the flow during 15 minutes. Then images were acquired with the Zeiss microscope. Graph represents Jurkat cell viability (%) in the device after HCQ exposure and correspond to two experiments (means ± SEM).

Figure S3.
